# Genome-wide identification and expression profiling of the *YUCCA* gene family in *Malus domestica*

**DOI:** 10.1038/s41598-020-66483-y

**Published:** 2020-07-02

**Authors:** Chunhui Song, Dong Zhang, Liwei Zheng, Yawen Shen, Xiya Zuo, Jiangping Mao, Yuan Meng, Haiqin Wu, Yike Zhang, Xiaoyuan Liu, Ming Qian, Jie Zhang, Gaochao Li, Caiping Zhao, Libo Xing, Juanjuan Ma, Mingyu Han, Na An

**Affiliations:** 1 College of Horticulture, Northwest A&F University, Yangling, Shaanxi 712100 China; 2 College of Horticulture, Henan Agriculture University, Zhengzhou, Henan 450002 China; 3 College of Life Sciences, Northwest A&F University, Yangling, Shaanxi 712100 China; 4 Gaotou Township People’s Government of Wuji County, Shijiazhuang Hebei, 050000 China; 5 Taiyuan Academy of Agricultural Sciences, Taiyuan, Shannxi 030000 China; 6 Weinan Agro-Tech Extension Center, Weinan, Shaanxi 714000 China; 7 Silkworm Tea Fruit Technology Extension Center in Nanzheng District, Hanzhong, 723100 Shaanxi China

**Keywords:** Plant sciences, Plant development, Plant hormones

## Abstract

The plant hormone auxin is essential for plant growth and development. YUCCA proteins catalyse the rate-limiting step for endogenous auxin biosynthesis. In this study, we isolated 20 *MdYUCCA* genes from apple genome. *MdYUCCA6a*, *MdYUCCA8a*, and *MdYUCCA10a* were expressed in most organs and could support whole plant basal auxin synthesis. *MdYUCCA4a*, *MdYUCCA10b*, and *MdYUCCA11a* expression indicated roles for these genes in auxin biosynthesis in vegetative organs. *MdYUCCA2b*, *MdYUCCA11b*, and *MdYUCCA11d* were mainly expressed in flower organs. High temperature induced the expression of *MdYUCCA4a*, *MdYUCCA6a*, *MdYUCCA8a*, and *MdYUCCA10a*, and down-regulated the expression of *MdYUCCA2b* and *MdYUCCA6b*. Dual-luciferase assay indicated that MdPIF4 could trans-activate the *MdYUCCA8a* promoter. Overexpression of *MdYUCCA8a* increased IAA content, increased stem height, enhanced apical dominance, and led to silique malformation. These results provide a foundation for further investigation of the biological functions of apple *MdYUCCAs*.

## Introduction

The plant hormone auxin is essential for plant growth and development, and plays important roles in biological processes, such as organ formation and development and apical dominance^1,2^. Auxin synthesis mainly occurs in young tissues, such as developing leaves, cotyledons, flowers, and shoots and root apex. Indole-3-acetic acid (IAA), the main auxin in plants, is *de novo* synthesized via tryptophan (Trp)-independent and Trp-dependent pathways, with synthesis of IAA from Trp proceeding independently via distinct pathway intermediates: indole-3-pyruvic acid (IPA), indole-3-acetaldoxime, indole-3-acetamide and tryptamine (TAM). The indole-3-pyruvic acid pathway is the primary Trp-dependent pathway for auxin biosynthesis and is conserved in plants. However, the physiological and molecular mechanism of the proposed Trp-independent pathway is less known. In the indole-3-pyruvic acid pathway, at the first step, Trp is converted into indole-3-pyruvate by tryptophan aminotransferase (TAA), then, YUCCA (YUC) flavin-containing monooxygenases catalyse the conversion of IPA to IAA^3,4^. The second step, catalysed by YUCCA, is a rate-limiting step for the indole-3-pyruvic acid pathway. The *YUCCA* family genes play a significant role in auxin-mediated developmental processes and have received much attention since their discovery.

YUCCA proteins contain several conserved motifs, including FAD-binding site, GC-motif, FMO-identifying sequence motif, NADPH-binding site and ATG-containing motif. Among these motifs, the FAD-binding site and NADPH-binding site are essential for YUCCA enzymatic activities. *Arabidopsis* YUCCAs also share low sequence similarity with thioredoxin (Trx) reductases (TrxR) from bacteria and plants and possess thiol-reductase (TR) activity^5^.

The *Arabidopsis YUCCA* gene family includes 11 members. Knockdown of any single *YUCCA* gene did not result in obvious abnormal phenotypes, whereas triple or quadruple *yucca* mutants showed severe defects in various developmental processes, suggesting that spatially and temporally regulated auxin biosynthesis involving multiple *YUCCA* genes is essential for plant development^2^. In *Arabidopsis*, YUC1, YUC2, YUC4, and YUC6 are the main YUC proteins functioned in shoots. Double mutants, *yuc1yuc4* and *yuc2yuc6*, all triple mutant combinations, and *yuc1yuc2yuc4yuc6* quadruple mutants had severe defects in vascular patterning and flower development^2^. Microspore development in *yuc2yuc6* double mutants was arrested before the first asymmetric mitotic division (PMI), and consequently, *yuc2yuc6* failed to produce viable pollen^6^. *YUC3*, *YUC5*, *YUC7*, *YUC8*, and *YUC9* were expressed during root development and *yuc3yuc5yuc7yuc8yuc9* quintuple mutants showed short and agravitropic root patterns^7^. *AtYUC10* and *AtYUC11* have been suggested to have overlapping functions with *AtYUC1* and *AtYUC4* during embryogenesis^1^. Similarly, in maize, a monocot-specific *YUCCA* like gene mutant *sparse inflorescence1*, showed defects in the formation of branches, spikelets, florets, and floral organs^8^. In rice, the *YUC* loss-of-function mutant *oscow1* demonstrated a reduced root-to-shoot ratio^9^. The petunia ortholog of *Arabidopsis YUC1* gene *FLOOZY* (*FZY*) was expressed in young leaves and developing flowers, which indicated that it was required for the formation of leaf secondary veins and flower architecture^10^. In the root parasitic plant *Phtheirospermum japonicum*, expression of *YUC3* at the epidermal cells near the contact site contributed to haustorium formation^11^. In peach, *PpYUC11* showed obvious tissue-specific expression during the mature climacteric stage of the melting flesh fruit and was involved in peach flesh texture development^12^. Overexpression of *YUCCA* genes resulted in auxin overproduction phenotypes, such as elongated hypocotyl; enhanced apical dominance; tall, slender stems; leaf hyponasty; changes in leaf shape; increased lateral root numbers; abnormal flower architecture; and sterility^7,13–15^.

Some *YUCCA* genes were also involved in plant responses to abiotic stress. High temperature increased IAA level and led to hypocotyl elongation in *Arabidopsis*^16^. The basic helix-loop-helix transcription factor PHYTOCHROME-INTERACTING FACTOR 4 (PIF4) regulated high temperature-induced hypocotyl elongation by activating *YUC8* in *Arabidopsis*^17,18^. RNA-binding protein FCA (Flowering time control protein) tuned down the high-temperature-induced stem elongation by modifying *YUC8* chromatin and dissociating PIF4 from the *YUC8* promoter^19^. In contrast, in barley and *Arabidopsis*, high temperature suppression of *YUC2* and *YUC6* gene expression in developing anthers resulted in male sterility, and exogenous IAA rescued the defects^20^. This indicated that the YUC-mediated local auxin biosynthesis in the anther was essential for male fertility. Furthermore, a 24-nt heterochromatic siRNA locus in the promoter of *YUC2* may contribute to epigenetic regulation of auxin homeostasis at ambient temperature^21^. Overexpression of *Arabidopsis YUCCA6* delayed dark-induced and hormone-induced leaf senescence in *Arabidopsis*^22^. Overexpression of *Arabidopsis YUCCA6* and *YUCCA7* enhanced resistance to water deficit^23,24^. Auxin concentrations were highly elevated under shade condition, and the expression of *YUC2*, *YUC5*, *YUC8*, and *YUC9* were up-regulated to enable shade avoidance induced by auxin increases^25^. Under aluminium stress, *YUCCA* regulated local auxin biosynthesis in the root apex transition zone, mediating root growth inhibition^26^.

Systematic identification of *YUCCA* genes has been performed in only a few plant species, such as *Arabidopsis*, strawberry, peach, poplar and cucumber^2,12,15,27^. Apple is a favourite fruit crop with high economic and nutritional value. In this study, we systematically identified 20 *YUCCA* family genes in apple genome, explored the expression of these *MdYUCCAs* in different tissues, and measured expression changes in response to high temperature. We also verified an interaction between the MdPIF4 protein and *MdYUCCA8a* promoter, and analysed the phenotype of *MdYUCCA8a* overexpression in *Arabidopsis*.

## Results

### Identification and classification of apple *MdYUCCA* genes

Using the *Arabidopsis* AtYUCCA BLAST homology search and conserved domain analysis, 20 *MdYUCCA* genes were identified in apple (Table 1). They were named based on their similarity to *Arabidopsis YUCCA* genes. Deduced amino acid lengths of these *MdYUCCAs* were between 338 and 563 aa. The pIs were between 8.56 and 9.88, and molecular weights were between 37.08 and 64.48 kD. These *MdYUCCA* genes were located on nine different chromosomes. Chromosome 10 had six *MdYUCCAs*. Chromosome 15 had five *MdYUCCAs*. Chromosome 8 contained two *MdYUCCAs*. Chromosome 1, 2, 3, 5, 13, and 16 each included one *MdYUCCA*. *MdYUCCA11a*, *MdYUCCA11b*, *MdYUCCA11d*, and *MdYUCCA11e* on Chromosome 10 appeared to result from tandem duplication.

**Table 1:** Characteristics of the *MdYUCCA* gene family in apple.

Gene name	GDDH13_1-1	NCBI	GDR v3.01	Location	Strand	Length (aa)	pI	MW
*MdYUCCA2a*	MD15G1184800	XM_008339168.1		Chr15:14580569..14584004	−	437	8.8	48.73
*MdYUCCA2b*	MD01G1067600	XM_008388239.1		Chr01:17093902..17096602	−	436	9.05	48.61
*MdYUCCA2c*	MD02G1046700			Chr02:3719433..3722141	−	381	8.96	42.55
*MdYUCCA3a*	MD16G1232600	XM_008344329.1		Chr16:23900387..23902351	+	426	9.08	47.69
*MdYUCCA3b*	MD13G1227000			Chr13:22205775..22207631	+	338	8.86	37.08
*MdYUCCA4a*	MD08G1139500	XM_008368432.1		Chr08:13470642..13472655	−	410	9.06	45.29
*MdYUCCA4b*	MD15G1117200	XM_008395801.1		Chr15:8337905..8340244	−	411	9.19	45.35
*MdYUCCA6a*	MD15G1098700	XM_008395672.1		Chr15:6833888..6838031	−	460	9.05	51.04
*MdYUCCA6b*	MD08G1119300	XM_008379894.1		Chr08:10869749..10873628	−	448	9.26	49.79
*MdYUCCA8a*	MD10G1172800	XM_008367718.1	MDP0000262982	Chr10:26514039..26515625	−	423	8.72	47.42
*MdYUCCA8b*	MD05G1184800			Chr05:31306814..31308517	−	423	8.72	47.37
*MdYUCCA10a*	MDP0000138851	XM_008370693.1	MDP0000138851	Chr03:31907169..31927564^a^	+	382	9.01	42.68
*MdYUCCA10b*	MD15G1133800			Chr15:9725184..9729059	−	563	9.88	64.48
*MdYUCCA10c*	MD00G1056000	XM_008370695.1	MDP0000208234	Chr00:10506121..10507905	+	381	8.96	42.50
*MdYUCCA11a*	MD10G1010300	XM_008366814.1	MDP0000259265	Chr10:1431009..1433012	+	383	8.78	43.01
*MdYUCCA11b*	MD10G1010000	XM_008380434.1	MDP0000582079	Chr10:1378296..1380232	+	380	8.56	41.98
*MdYUCCA11c*	MD15G1274600	MDP0000686821	MDP0000686821	Chr15:24088780..24090298	−	385	9.09	42.85
*MdYUCCA11d*	MD10G1010200			Chr10:1404169..1405726	+	365	8.96	40.67
*MdYUCCA11e*	MD10G1010100			Chr10:1386074..1387630	+	365	8.96	40.67
*MdYUCCA11f*	MDP0000209681	XM_008350515.1	MDP0000209681	Chr10:956025..957926^a^	−	365	8.88	40.65

^a^Chromosome location at the GDR v3.01 genome. The chromosome location information of the other *MdYUCCAs* was from the GDDH13_1-1 version genome.

### Phylogenetic analysis of MdYUCCA and exon distribution

**Figure 1:**
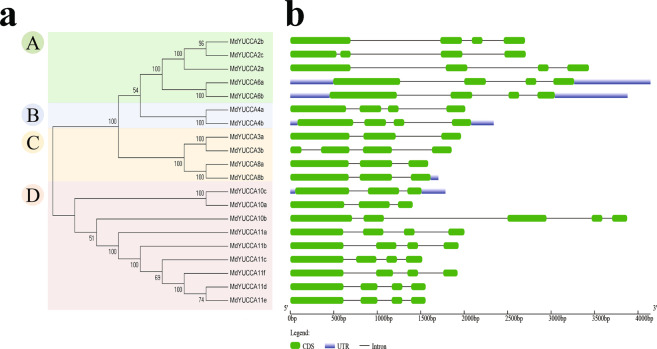
Phylogenetic tree and gene structure of *MdYUCCA*. (**a**) Phylogenetic analysis of MdYUCCA, constructed with the neighbor-joining method in MEGA 7.0 software. A, B, C, and D represent different gene subfamilies. (**b**) Structure of *MdYUCCA* genes. Intron, exon and untranslation region (UTR) are represented by black lines, green boxes and light blue boxes respectively.

MdYUCCAs were divided into four subfamilies based on their similarity. MdYUCCA2s and MdYUCCA6s were in the subfamily A. MdYUCCA4s were in the subfamily B. MdYUCCA3s and MdYUCCA8s were in the subfamily C. MdYUCCA10s and MdYUCCA11s were in the subfamily D (Fig. 1a). *MdYUCCA10b* contained five exons. *MdYUCCA3a*, *MdYUCCA8a*, *MdYUCCA8b*, *MdYUCCA10a* and *MdYUCCA10c* contained three exons. The other *MdYUCCA* genes contained four exons (Fig. 1b).

**Figure 2:**
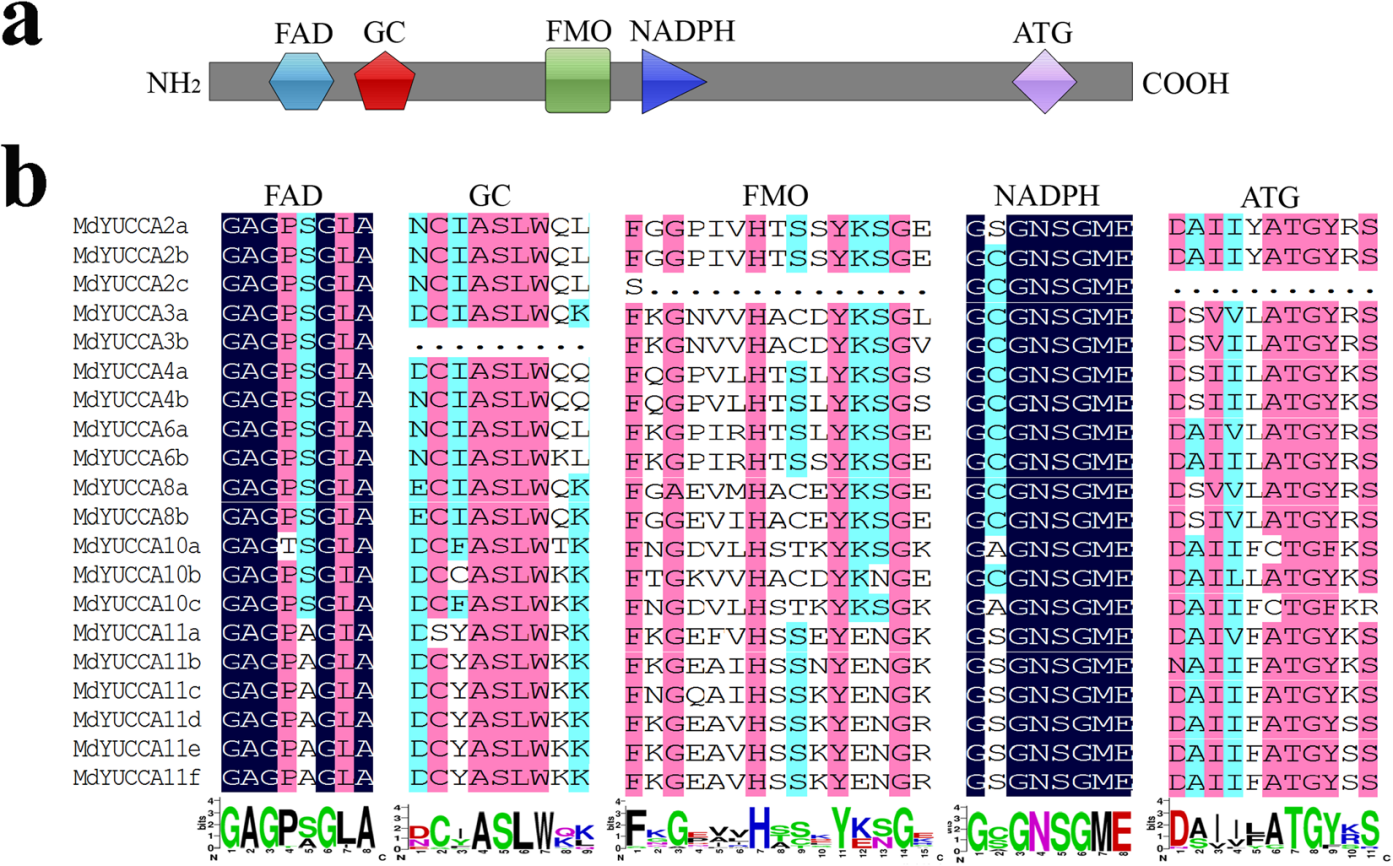
Conserved domains in MdYUCCA. (**a**) Conserved domains of MdYUCCA. (**b**) Alignment of conserved domains in MdYUCCA.

Conserved domain analysis revealed that most MdYUCCAs included five conserved motifs: FAD-binding domain, GC-motif, FMO-identifying sequence, NADPH domain, and ATG-containing motif (Fig. 2a). MdYUCCA2c lacked the FMO-identifying sequence and ATG-containing motif. MdYUCCA3b lacked the GC-motif (Fig. 2b).

### Evolutionary relationship of YUCCAs

**Figure 3:**
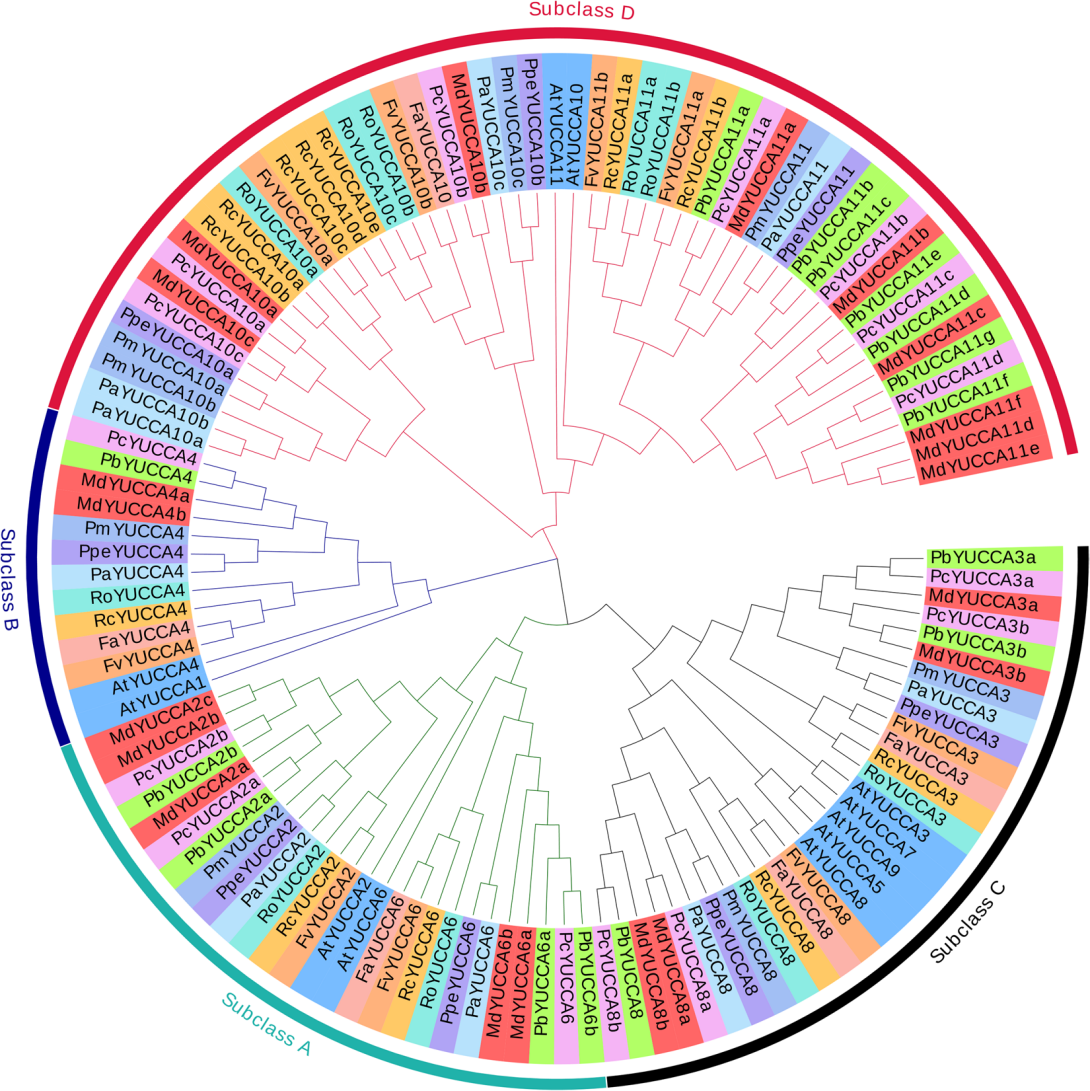
Phylogenetic tree of YUCCA from *Rosaceae* species. At: *Arabidopsis thaliana*; Md: *Malus domestica*; Fv: *Fragaria vesca*; Fa: *Fragaria ananassa*; Ppe: *Prunus persica*; Pb: *Pyrus bretschneideri*; Pc: *Pyrus communis*; Rc: *Rosa chinensis*; Pm: *Prunus mume*; Ro: *Rubus occidentalis*.

To describe the evolutionary relationship of YUCCA genes in *Rosaceae*, MdYUCCAs were compared with YUCCA genes from *Arabidopsis* (as reference), peach, European pear, white pear, wood strawberry, strawberry, sweet cherry, rose, and rubus (Fig. 3). *Rosaceae* YUCCAs were divided into four subclasses based on their similarity. MdYUCCA2s and MdYUCCA6s were placed in subclass A. MdYUCCA4s comprised subclass B. MdYUCCA3s and MdYUCCA8s were included in subclass C. Nine MdYUCCA genes, including MdYUCCA10s and MdYUCCA11s comprised subclass D. There were 20 MdYUCCA genes identified in apple, more than in other studied *Rosaceae* species. Homologs of *Arabidopsis* YUCCA1, YUCCA5, YUCCA7, and YUCCA9 were not found in apple and other *Rosaceae* species. YUCCA10 and YUCCA11 were expanded in some *Rosaceae* species. For example, there were six and seven YUCCA11 homologs in apple and white pear, respectively.

**Figure 4:**
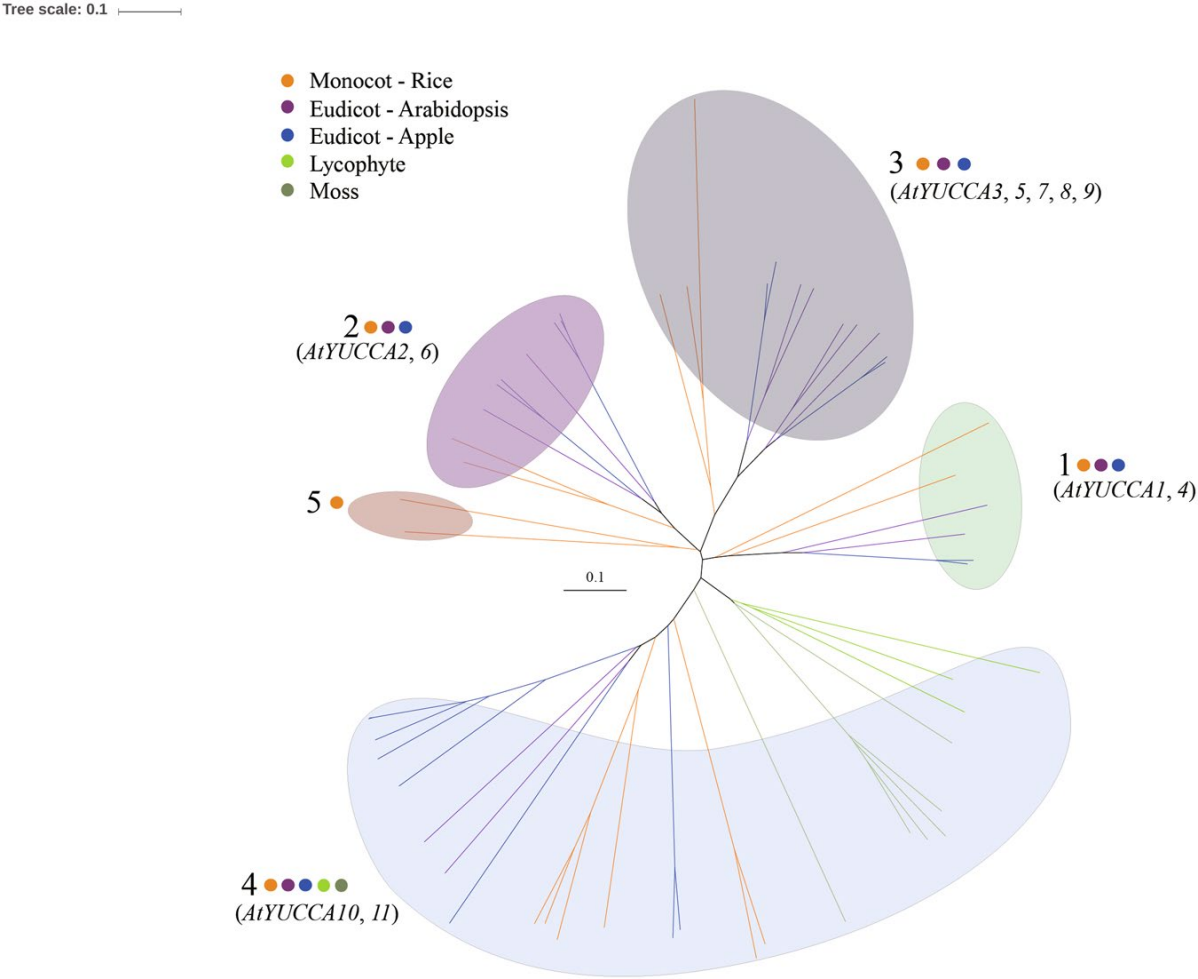
Phylogenetic relationships of YUCCAs in vascular plants, moss, and algae. The bubbles indicate the five YUCCA subgroups. The dots symbolize the species to which the YUCCA proteins in each group belong (brown: rice [monocot]; purple: *Arabidopsis thaliana* [eudicot]; cyan-blue: apple [eudicot]; green: *Selaginella moellendorffii* [lycophyte]; and dark green: *Physcomitrella patens* [moss]).

To explore the evolution of *YUCCA* genes, a phylogenetic tree was constructed with YUCCA genes from six species: a monocot, two eudicots, a lycophyte, a moss, and an algae. As shown in Fig. 4, 55 YUCCA proteins were classified into five subgroups based on clades and topology in the tree. The 20 apple YUCCAs, together with 11 AtYUCCAs and 13 OsYUCCAs, were distributed into subgroup 1, 2, 3, and 4. Two OsYUCCAs were placed in subgroup 5. YUCCAs from lycophyte and moss were only placed in subgroup 4, which included YUCCA10 and YUCCA11. There were no YUCCA homologs detected in the algae *Chlamydomonas reinhardtii*.

### Expression pattern of *MdYUCCA* genes in different tissues

**Figure 5:**
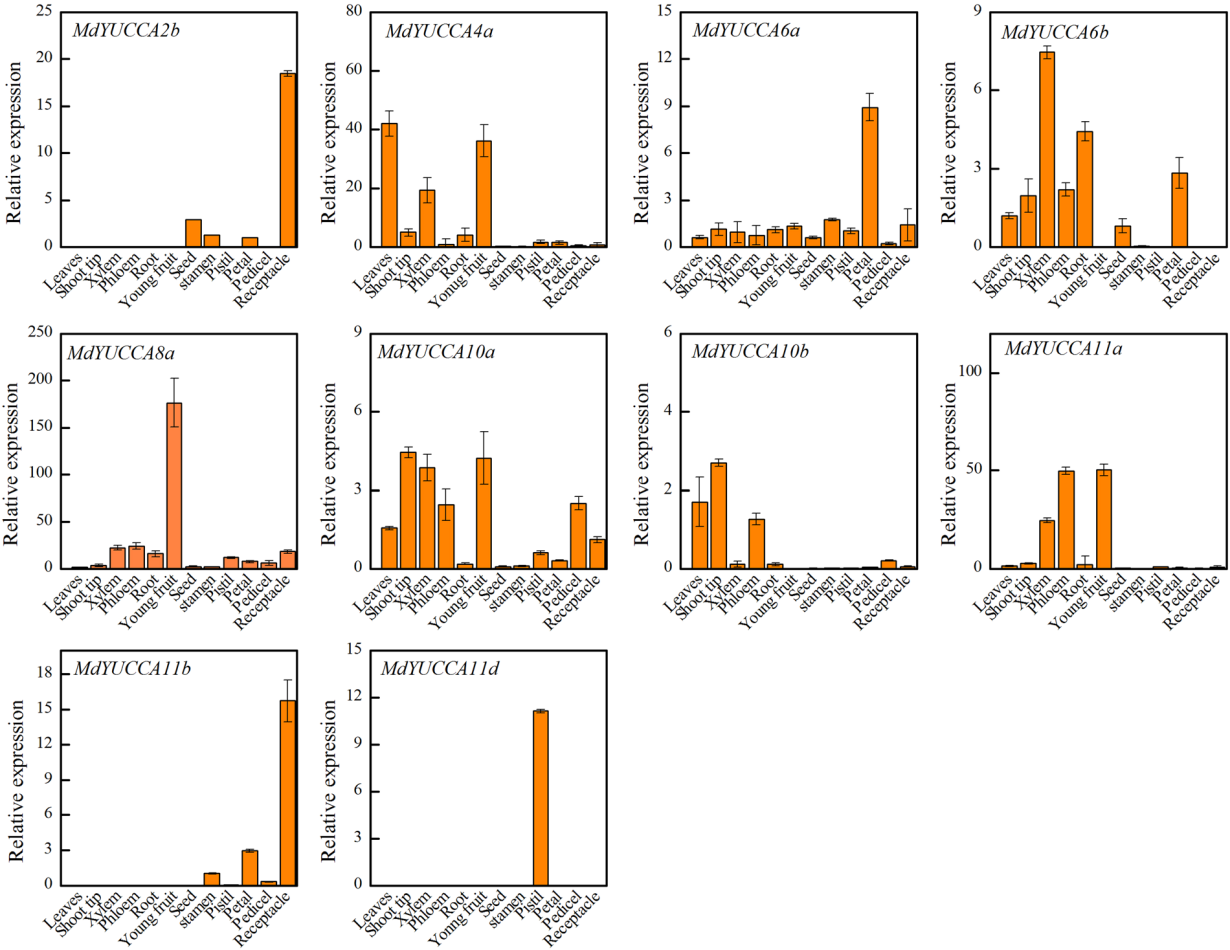
Expression of *MdYUCCAs* in different tissues. Gene expression of *MdYUCCAs* was measured in leaves, shoot tip, xylem, phloem, root, young fruit, seed, stamen, pistil, petal, pedicel, and receptacle. The *MdACTIN* gene and *MdEF* were used as the internal control to normalize the real-time PCR data. Error bars indicate SEs (standard errors) from 3 biological repetitions.

To explore *MdYUCCA* expression patterns in different tissues, real-time RT-PCR was used to measure transcript levels in the leaves, shoot tip, xylem, phloem, root, young fruit, seed, stamen, pistil, petal, pedicel, and receptacle of 4-year-old apple trees (Fig. 5). *MdYUCCA2b* was mainly expressed in the flower organ, with the highest expression in the receptacle. *MdYUCCA4a* was mainly expressed in the leaves, xylem, and young fruit. *MdYUCCA6a* was detected in all examined tissues and showed high expression in petal. *MdYUCCA6b* was highly expressed in xylem, root, and petal. *MdYUCCA8a* was highly expressed in young fruit. *MdYUCCA10a* was highly expressed in the shoot tip, young fruit, and xylem. *MdYUCCA10b* showed high expression in shoot tip. *MdYUCCA11a* was highly expressed in the phloem and young fruit. *MdYUCCA11b* showed high expression in receptacle. *MdYUCCA11d* was only expressed in the pistil.

### YUCCA enzyme inhibitor Yucasin decreased flower petal width

**Figure 6:**
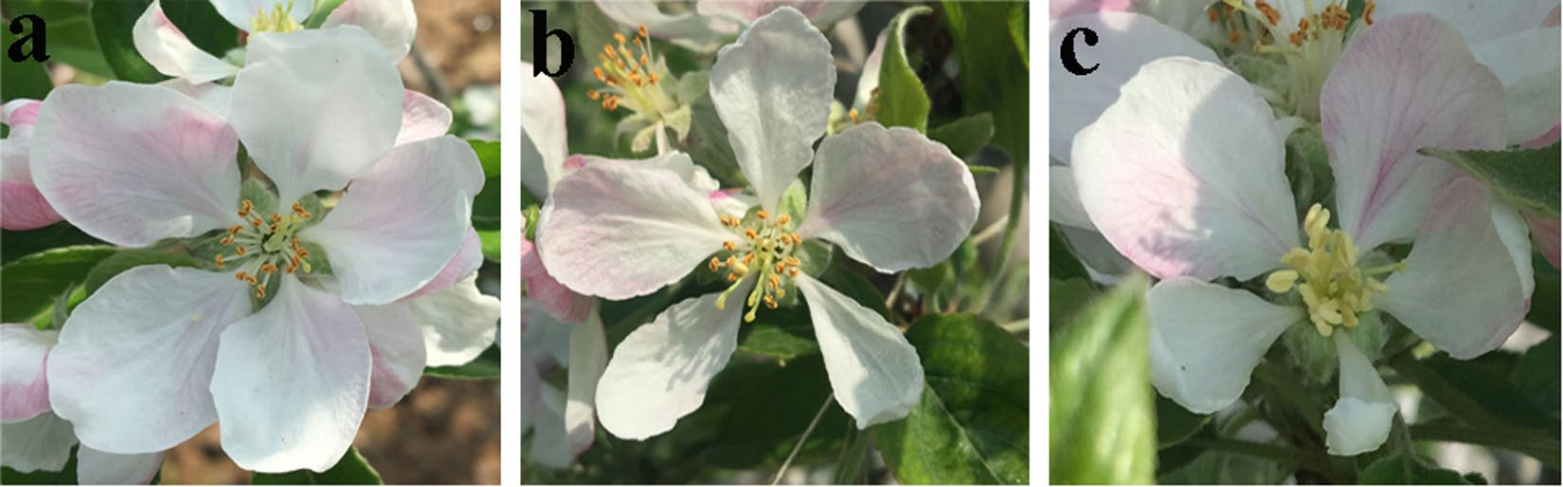
YUCCA enzyme inhibitor Yucasin decreased flower petal width. (**a**) Control, the flower buds were injected with the sterile water. (**b**,**c**) Yucasin treatment. The flower buds were injected with 300 μL 100 μM Yucasin.

*MdYUCCAs* gene expression revealed that some *MdYUCCAs* were highly expressed in floral organs. Auxin plays important roles in floral organ development^2,28^. Yucasin is an effective competitive inhibitor of the YUCCA enzyme^13^. Thus, Yucasin was used to determine if inhibition of YUCCA activity affected flower organ development. The results showed that injecting Yucasin into dormant apple flower buds significantly decreased flower petal width (Fig. 6).

### High temperature regulated the expression of *MdYUCCAs*

**Figure 7:**
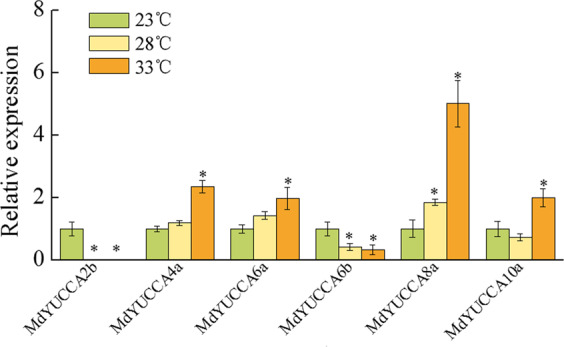
Effect of temperature on the expression of *MdYUCCA*s. The asterisks indicated statistical significance (*p* < 0.05) compared with the control (23 °C). Error bars indicate SEs from 3 biological repetitions.

High temperature led to auxin-mediated hypocotyl elongation in *Arabidopsis thaliana*^17,18^. In apple, high temperature significantly increased *MdYUCCA4a*, *MdYUCCA6a*, *MdYUCCA8a*, and *MdYUCCA10a* expression, but down-regulated the expression of *MdYUCCA2b* and *MdYUCCA6b*. Expression of *MdYUCCA8a* was markedly induced by high temperature, with expression at 33 °C and 28 °C that was 5.0 and 1.8 times higher than at 23 °C, respectively (Fig. 7).

### Activity of *MdYUCCA8a* and *MdYUCCA10a* promoters in response to hormone treatment

**Figure 8:**
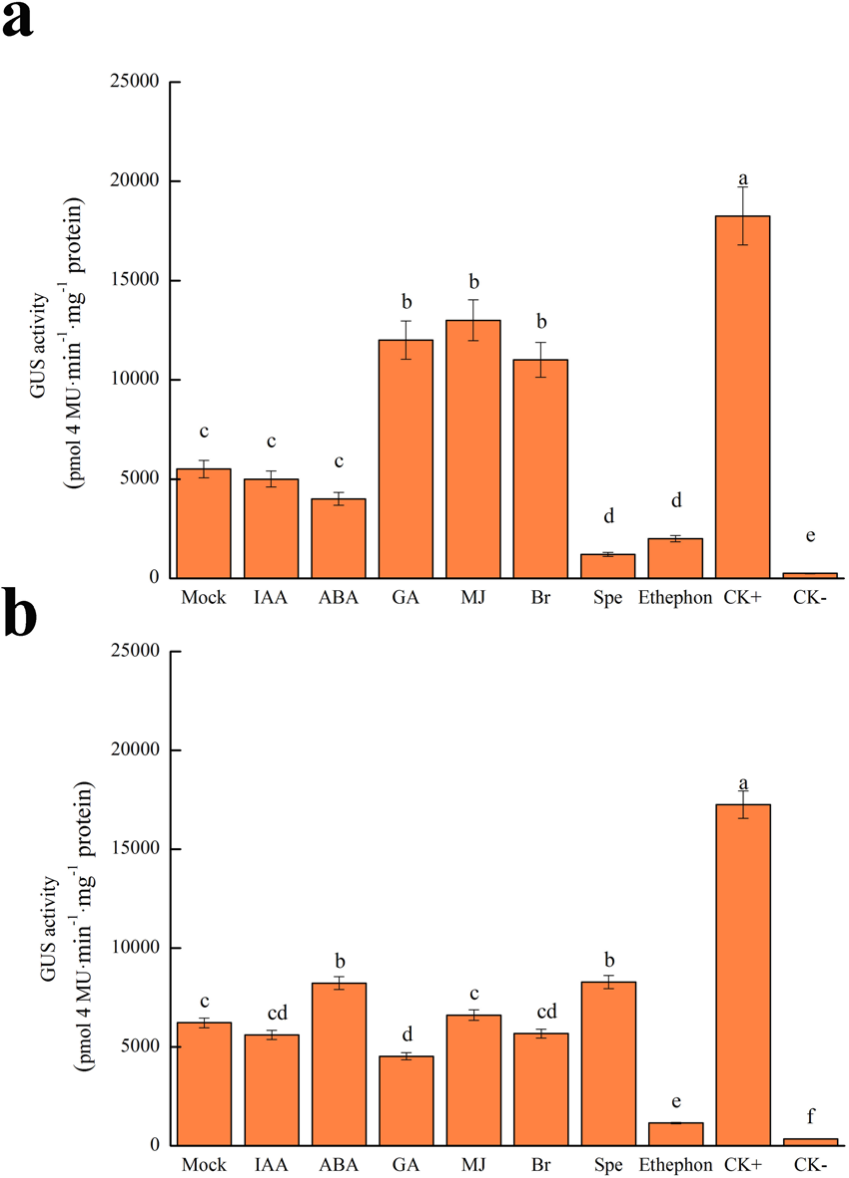
GUS activity of *MdYUCCA8a* and *MdYUCCA10a* promoters in response to hormone treatment. (**a**) GUS activity of transiently transforming the tobacco leaves by *MdYUCCA8a* promoter. (**b**) GUS activity of transiently transforming the tobacco leaves by *MdYUCCA10a* promoter. Mock: inoculation of MdYUCCApro-pCAMBIA1381-GUS bacterium solution and then spraying water, IAA: indole-3-acetic acids; ABA: abscisic acid; GA: gibberellin 3; MJ: methyl jasmonate; Br: brassinolide; Spe: spermidine; CK + : positive control, inoculation of 35S-pCAMBIA1381-GUS bacterium solution and then spraying water; CK−: negative control, inoculation of pCAMBIA1381-GUS bacterium solution and then spraying water. Error bars indicate SEs from three biological replications. Statistically significant differences are indicated by different lower case letters (*p* < 0.05, one-way ANOVA).

We used a GUS reporter to measure *MdYUCCA8a* and *MdYUCCA10a* promoter activity in tobacco leaves under various hormone treatment (Fig. 8). The *MdYUCCA8a* promoter activity was induced 2.18, 2.36, and 2.00 times over that of mock treatment by gibberellic acid, methyl jasmonate, and brassinolide, respectively. Spermidine (a polyamine) and ethephon (a synthetic compound which decomposes into ethylene) significantly inhibited *MdYUCCA8a* promoter activity (Fig. 8a). The *MdYUCCA10a* promoter was induced 1.32 and 1.33 times over that of mock treatment by ABA and spermidine, respectively. Ethephon significantly inhibited *MdYUCCA10a* promoter activity (Fig. 8b).

### Interaction of transcription factor MdPIF4 with the *MdYUCCA8a* promoter

**Figure 9:**
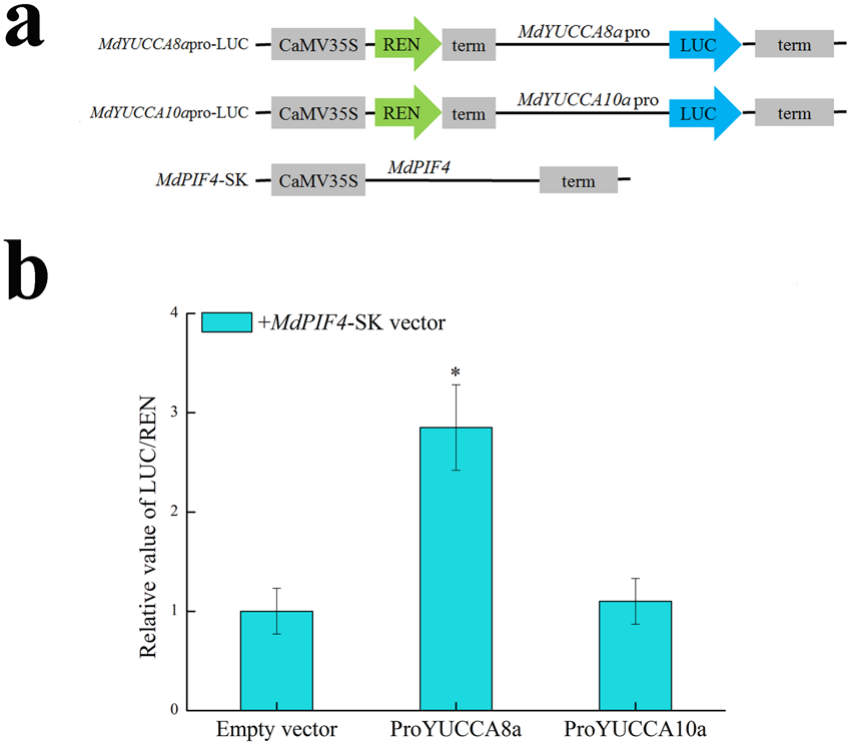
Dual luciferase transient expression assay to probe interaction of transcription factor MdPIF4 and *MdYUCCA8a* promoter in tobacco leaves. (**a**) Vector construction diagram. (**b**) The activity of firefly and renilla luciferase in tobacco leaves was measured 3 d after infiltration. Error bars show SEs of three independent experiments with at least four replicate reactions. The asterisks indicate statistical significance (*p* < 0.05).

PIF4 binds to the *YUCCA* promoter and promotes YUCCA expression and auxin synthesis^18^. The dual luciferase transient expression system was used to determine if there was an interaction between MdPIF4 and the *MdYUCCA8a* promoter in tobacco leaves. A 2.8-fold enhancement of activity in the dual-luciferase assay indicated that MdPIF4 can trans-activate the *MdYUCCA8a* promoter (Fig. 9). Although the *MdYUCCA10a* promoter also contained G-box elements, we did not observe MdPIF4 activation of the *MdYUCCA10a* promoter.

### The phenotype of plants overexpressing *MdYUCCA8a*

**Figure 10:**
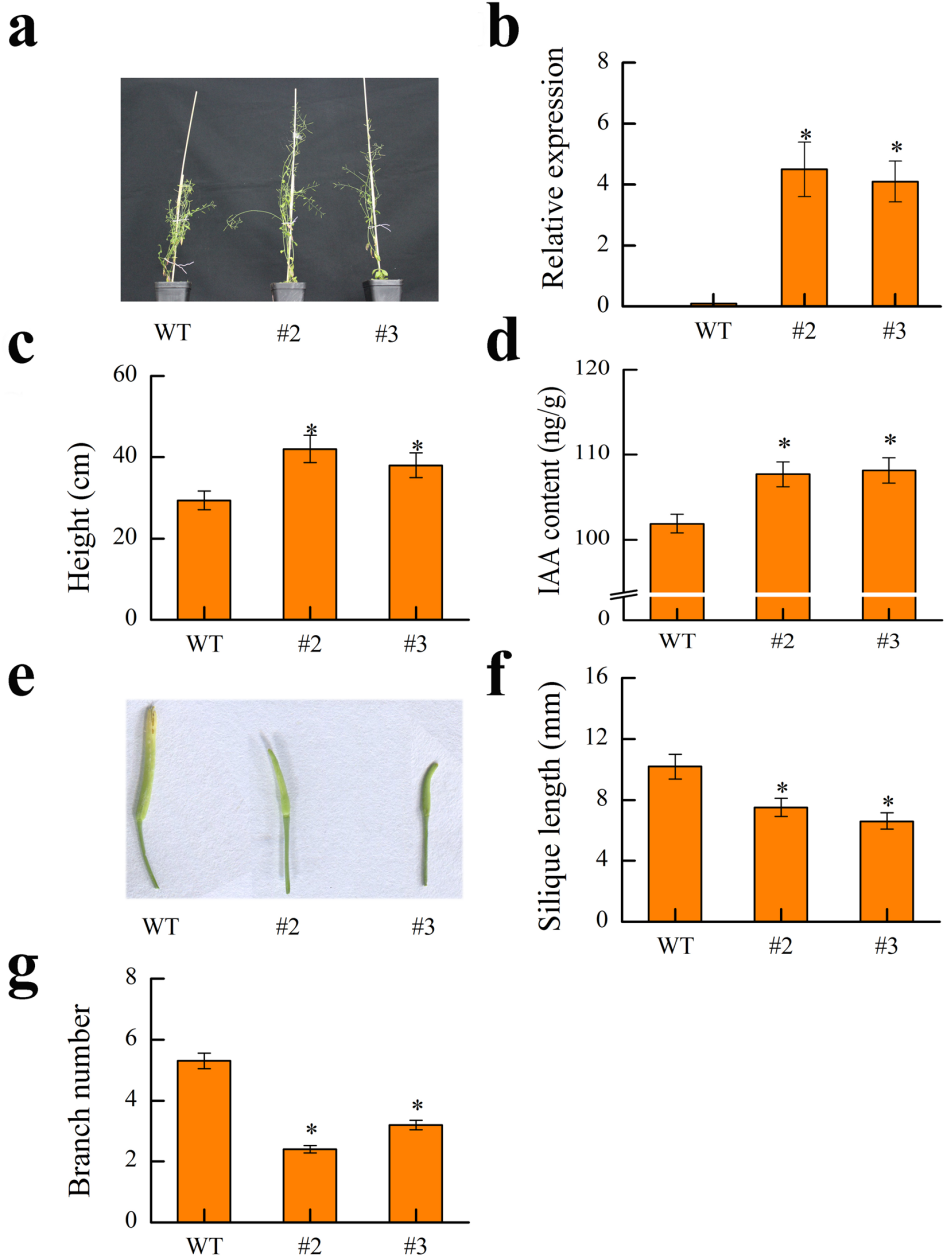
Phenotype of *MdYUCCA8a* overexpression in *Arabidopsis thaliana*. (**a**) Photos of wild type (WT) and overexpressing *MdYUCCA8a* gene (#2 and #3) *Arabidopsis thaliana*. (**b**) qRT-PCR analysis of *MdYUCCA8a* expression in WT and transgenic lines. *Arabidopsis thaliana AtACTIN2* was used as the reference gene. (**c**) Plant height. (**d**) IAA content. (**e**) Photos of siliques. (**f**) Siliques length. (**g**) Branch number. The asterisks indicate statistical significance (*p* < 0.05). Error bars indicate SEs from three replications.

*MdYUCCA8a* was ectopically expressed in wild type Columbia ecotype *Arabidopsis thaliana* with a constitutive 35S strong promoter. Eight independent T3 generation transgenic lines were obtained, and line #2 and #3 were selected for phenotypic analysis (Fig. 10). Overexpression of *MdYUCCA8a* increased stem height, as the height of line #2 and #3 were 42% and 29% higher than that of wild type, respectively (Fig. 10a,c). The transgenic lines showed high IAA content (Fig. 10d). Overexpression of *MdYUCCA8a* led to silique malformation and the silique length was significantly reduced (Fig. 10e,f). Apical dominance significantly increased in the transgenic lines, as the number of lateral shoots in overexpression lines was significantly lower than in wild-type (Fig. 10g).

## Discussion

YUCCA is a rate-limiting enzyme in the indole-3-pyruvate (IPA) pathway and converts IPA into IAA. In this study, we identified 20 *MdYUCCA* genes in apple. Phylogenetic analysis shown *MdYUCCA* genes could be divided into four subfamilies. Homologs of *Arabidopsis YUCCA1*, *YUCCA5*, *YUCCA7*, and *YUCCA9* were not found in *Rosaceae* species and cucumber^15^. These *YUCCA* genes may have been lost via chromosomal fragment deletion. *YUCCA11* was expanded in apple and white pear, which contained six and seven *YUCCA11* members, respectively. There has been a tandem duplication of *MdYUCCA11s* in Chromosome 10.

Auxin biosynthesis occurs both at local auxin response sites and at distant sites as a source for polar auxin transport. Auxin functions may be dependent on the location of auxin production. Plants use different *YUCCA* genes for auxin biosynthesis in different organs. In apple, *MdYUCCA6a*, *MdYUCCA8a*, and *MdYUCCA10a* were expressed in most organs and may be involved in whole plant basal auxin synthesis. *MdYUCCA4a*, *MdYUCCA10b*, and *MdYUCCA11a* were the main *YUCCA* genes for auxin biosynthesis in vegetative organs. *MdYUCCA2b*, *MdYUCCA11b*, and *MdYUCCA11d* may be responsible for auxin synthesis in flower organs. In other plants, the expression of certain *YUCCA* in specific tissues has indicated involvement in the development of specific organs. Peach *PpYUCCA11* transcripts specifically accumulated during the late ripening stage in melting flesh peaches, suggesting involvement in fruit softening^12^. In cucumber, *CsYUCCA11* uniquely accumulated in the opened male flower^15^. In apple, *MdYUCCA11d* appeared to be specifically expressed in the pistil, suggesting that it may be responsible for auxin synthesis there. *YUCCA11* seem to have specific roles in flower and fruit development.

High temperature has been shown to induce elevated levels of endogenous free indole-3-acetic acid (IAA). In this study, high temperature induced the expression of *MdYUCCA4a*, *MdYUCCA6a*, *MdYUCCA8a*, and *MdYUCCA10a*. Dual-luciferase assay results showed that MdPIF4 could bind to G-box CACGTG DNA fragments in the promoter of *MdYUCCA8a*, but not *MdYUCCA10a*. This is in accordance with previous results in *Arabidopsis* that showed PIF4 directly binds to the *YUCCA8* promoter, but not to other YUCCA promoters, with preferential binding of the CACGTG G-box motif^18,29^. PIF4-activated expression of *YUCCA8* may be a conserved molecular mechanism that elevates endogenous IAA level under high temperature conditions. High temperature may regulate other YUCCA genes through indirect or PIF4-independent pathways. High temperature down-regulated the expression of *MdYUCCA2b* and *MdYUCCA6b*. In barley and *Arabidopsis*, high temperature conditions caused male sterility by repressing *YUCCA2* and *YUCCA6* expression, which decreased auxin levels in the developing anthers^20^. Thus, high temperature has varying up-regulation and down-regulation effects on different *YUCCA* genes in the different biological process.

Overexpression of *MdYUCCA8a* in *Arabidopsis* increased the free auxin level and produced expected auxin overproduction phenotypes, like taller plant height and enhanced apical dominance, but also induced silique malformation. In previous studies, *YUCCA* transgenic lines also showed reproductive organ defects. Overexpression of strawberry *FaYUC1*, wheat *TaYUC10*, and cucumber *CsYUC11* in *Arabidopsis* resulted in defective pollen and sterility^15,30,31^. Overexpression of *FvYUC6* in woodland strawberry also delayed flowering and led to severe male sterility^32^. Repression of *YUCCA2* and *YUCCA6* expression by high temperature stress decreased auxin content and caused male sterility in barley and *Arabidopsis*^20^. Hence, appropriate auxin content is necessary for reproductive organ development.

In conclusion, we have systematically identified 20 *MdYUCCAs* in apple. Gene expression results suggested that *MdYUCCA6a*, *MdYUCCA8a*, and *MdYUCCA10a* were expressed in most organs and could support whole plant basal auxin synthesis. *MdYUCCA4a*, *MdYUCCA10b*, and *MdYUCCA11a* were the main *YUCCA* genes for auxin biosynthesis in vegetative organs. *MdYUCCA2b*, *MdYUCCA11b*, and *MdYUCCA11d* could be mainly responsible for auxin synthesis in flower organs. High temperature induced expression of *MdYUCCA4a*, *MdYUCCA6a*, *MdYUCCA8a*, and *MdYUCCA10a* and down-regulated expression of *MdYUCCA2b* and *MdYUCCA6b*. MdPIF4 interacted with the *MdYUCCA8a* promoter. Overexpression of *MdYUCCA8a* increased IAA content, increased stem height, enhanced apical dominance, and led to silique malformation.

## Methods

### Identification of *MdYUCCA* genes in the apple genome

*Arabidopsis* AtYUCCA protein sequences retrieved from TAIR (The Arabidopsis Information Resource: http://www.arabidopsis.org/) were separately used as blastp queries against apple genome GDR v3.01 (downloaded from the Genome Database for Rosaceae, http://www.rosaceae.org), the protein sequence of *Malus domestica* from NCBI (https://www.ncbi.nlm.nih.gov), and the apple genome GDDH13_1-1 (https://iris.angers.inra.fr/gddh13/the-apple-genome-downloads.html) using a stand-alone version of BLAST (Basic Local Alignment Search Tool: http://blast.ncbi.nlm.nih.gov)^33^. Similar sequences with e-values <0.0001 were further inspected for FMO-like and NAD binding domains using the domain analysis programs Pfam (Protein family: http://pfam.sanger.ac.uk/)^34^ and SMART (Simple Modular Architecture Research Tool: http://smart.embl-heidelberg.de/)^35^ with default cutoff parameters. After combining the predicted proteins and eliminating redundancy, the remaining peptide sequences were considered MdYUCCA candidates. Redundant *MdYUCCA* genes isolated from different apple reference genomes were identified by multiple sequence alignment and from their genome location. ExPASY (http://www.expasy.org/tools/) was used to predict the isoelectric point (pI) and molecular weight (MW) of each MdYUCCA. Exon/intron structure was determined with alignments of coding and genomic sequences and depicted with the online tool GSDS (http://gsds.cbi.pku.edu.cn/).

### Multiple sequence alignment, phylogenetic analysis, and classification of apple *MdYUCCA* genes

To establish the evolutionary relationship of MdYUCCAs to other *Rosaceae* YUCCA proteins, MdYUCCA protein sequences were compared with YUCCA sequences from *Arabidopsis thaliana* (as a reference), peach (*Prunus persica* L. Batsch), European pear (*Pyrus communis*), white pear (*Pyrus bretschneideri* Rehd.), woodland strawberry (*Fragaria vesca*), strawberry (*Fragaria ananassa*), sweet cherry (*Prunus avium*), rosa (*Rosa chinensis*), black raspberry (*Rubus occidentalis*), and mei (*Prunus Mume*).

To investigate YUCCA evolution in species ranging from algae to land plants, a phylogenetic tree was constructed with YUCCA proteins from six species (monocot-*Oryza sativa*, eudicots-apple and *Arabidopsis*, lycophyte-*Selaginella moellendorffii*, moss-*Physcomitrella patens*, and algae-*Chlamydomonas reinhardtii*). The phylogenetic tree was constructed using MEGA 7.0 with the neighbor-joining (NJ) method, 1000 bootstrap replicates, and complete delete parameters.

### Plant materials and treatment

Leaves, shoot tip, xylem, phloem, root, young fruit, seed, stamen, pistil, petal, pedicel, and receptacle were sampled from 4-year-old Fuji apple trees. Trees were located in experimental plots of the Yangling Subsidiary Center at the National Apple Improvement Center, Yangling, China (34.31°N, 108.04°E), and were trained and managed with standard horticultural practices.

For the temperature treatment, 1-year-old apple trees were grown in the growth chambers for 4 weeks under 12 h/12 h (light/dark) daily cycle at 22 °C, 28 °C, or 33 °C during the daytime, and at 18 °C at night. Shoot tips were harvested for RNA extraction and gene expression analysis.

For YUCCA inhibition experiments, 300 μL of 100 μM Yucasin, a YUCCA enzyme inhibitor (cat. 573760, Sigma, USA), were injected into dormant Fuji flower buds in mid-November with an injection syringe. The control was injected with sterile water. Apple flower phenotype was assessed at the full-bloom stage of the second year.

### RNA extraction, cDNA synthesis, and quantitative RT-PCR gene expression analysis

Total RNA was extracted from frozen samples according to the cetyltrimethylammonium bromide (CTAB) method^36^. The first-strand cDNA was synthesized with 1 µg of total RNA in a volume of 20 µL using reverse transcriptase. Quantitative reverse transcription-polymerase chain reaction (qRT-PCR) was used to analyze the gene expression. PCR amplification conditions was as follows: 95 °C for 5 min for initial denaturation, then 45 cycles of 94 °C for 20 s, 60 °C for 20 s (determined by the primer), and 72 °C for 10 s. Fluorescence was measured at the end of each cycle. Primer sequences for the quantification of transcripts by RT-PCR are listed in Supplemental Table S1. *MdActin* and *MdEF* were used as internal references for apple gene expression. Gene relative expression was calculated using the comparative 2^−∆∆CT^ method^37^.

### The dual-luciferase assay

The promoter sequences of *MdYUCCA8a* and *MdYUCCA10a* were ligated into pGreenII_0800-5_LUC vector. The CDS sequence of transcription factor *MdPIF4* was ligated into pGreenII_0029_62-SK vector. The recombinant plasmids were transformed into *Agrobacterium* GV3101. Dual-luciferase transient expression in tobacco leaves was performed in accordance with Hellens’ method^38^. Primer information is listed in Supplemental Table S1.

### Promoter activity assay

About 2000 bp promoter fragments from *MdYUCCA8a* and *MdYUCCA10a* were each cloned from Fuji genomic DNA. The promoter fragments were ligated into the pCAMBIA1381-GUS plant transformation vector with a ClonExpress II kit (Vazyme Biotech Co., Ltd, China). Primer information is listed in Supplemental Table S1. The recombinant plasmids were transformed into *Agrobacterium* strain LBA4404 and then transformed into leaf epidermal cells of 4-week-old tobacco (*Nicotiana Benthamiana*) plants by syringe infiltration. Next, transfected leaves were sprayed with different hormones respectively, including 100 μM indole-3-acetic acids, 100 μM ABA, 100 μΜ GA_3,_ 100 μΜ methyl jasmonate, 0.416 μΜ brassinolide, 100 μΜ spermidine, 3.46 mM ethephon. The GUS activity assay was performed as described by Jefferson *et al*.^39^.

### Construction of *MdYUCCA8a* overexpression vector and *Arabidopsis* transformation

The full CDS of *MdYUCCA8a* was cloned from Fuji apple and ligated into pCAMBIA1301 vector with a ClonExpress II kit (Vazyme Biotech Co., Ltd, China). Primer information is listed in Supplemental Table S1. Recombinant plasmids were transformed into *Agrobacterium* strain LBA4404. *MdYUCCA8a* was introduced into the wild type (WT) Columbia ecotype *Arabidopsis* (Col-0) using the floral dip method^40^. Seeds from positive transgenic plants were harvested individually. T3 generation homozygous transgenic lines were used for further investigation. Four weeks old rosette leaves were used to measure IAA and for RNA extraction and gene expression analysis. *AtActin2* gene was used as internal references. The IAA was extracted using solid-phase extraction methods, quantifed by the High Pressure Liquid Chromatography (Waters, USA) and enzyme-linked immunosorbent assay^41–43^.

## Supplementary information

Supplementary information.
