# Supplementary material for: Genome-wide identification and expression profiling of the YUCCA gene family in Malus domestica
Source: Sci Rep. 2020 Jul 2;10:10866. doi: 10.1038/s41598-020-66483-y (PMC7331580; doi:10.1038/s41598-020-66483-y)
Supplement: Supplementary file 1 — Supplementary information. [file 41598_2020_66483_MOESM1_ESM.docx]

**Supplementary Information**

**Genome-wide identification and expression profiling of the *YUCCA* gene family in *Malus domestica***

Chunhui Song^a,b,1^, Dong Zhang^a,1^, Liwei Zheng^a^, Yawen Shen^a^, Xiya Zuo^a^, Jiangping Mao^a^, Yuan Meng^a^, Haiqin Wu^d^, Yike Zhang^e^, Xiaoyuan Liu^f^, Ming Qian^a^, Jie Zhang^g^, Gaochao Li^a^, Caiping Zhao^a^, Libo Xing^a^, Juanjuan Ma^a^ , Mingyu Han^a^ & Na An^a,c,^*

Table S1 Primers sequences

| Gene | Forward primer (5'-3') | Reverse primer (5'-3') |  |
| --- | --- | --- | --- |
| *MdYUCCA2b* | TTGGTGGTTGGATGTGGTA | ACAGGAGTCTTGCCAGTC | RT-qPCR |
| *MdYUCCA4a* | TAGGCAACACAGACCAATTAGG | ACAATGCTCCAACATCAAGAAC | RT-qPCR |
| *MdYUCCA6a* | GGACTACTTGGTGCCTCAATGGA | ATGATGGTGGTGATGATGATGATAGTG | RT-qPCR |
| *MdYUCCA6b* | GCCAGGAAACATTTTACCCAA | CACCGTTTTCACTTCATGC | RT-qPCR |
| *MdYUCCA8a* | TCTCTTCCGCTTATTATTG | CATATAGGTCCACTAACAC | RT-qPCR |
| *MdYUCCA10a* | CAAGTATCCGATCATTGAC | CCTCTTATGCTGCCTATT | RT-qPCR |
| *MdYUCCA10b* | ACACCAGTTTATCCAGTA | TTCACAGTCCATTTCTTG | RT-qPCR |
| *MdYUCCA11a* | CATTGTAGGTGCCGGTCCA | AGCTGTTTCCACAACTCGGTC | RT-qPCR |
| *MdYUCCA11b* | ACTTGACAAAGCCACCTAACCAA | TGTAGGTGTATCCGAAGG | RT-qPCR |
| *MdYUCCA11d* | TCGGGATTCGAAGGCCAAAA | TTCGTGGCATTCCATTGTCATC | RT-qPCR |
| *MdActin* | TGACCGAATGAGCAAGGAAATTACT | TACTCAGCTTTGGCAATCCACATC | RT-qPCR |
| *MdEF* | ATTCAAGTATGCCTGGGTGC | CAGTCAGCCTGTGATGTTCC | RT-qPCR |
| *AtActin2* | AGTGTCTGGATCGGTGGTTC | CCCAGCTTTTTAAGCCTTT | RT-qPCR |
| MdPIF4a-CDS-SK | cgggatccATGAATTCTTGCATTCCCGAG | ggggtaccACTCATTTTTCCACTTAA | Clone |
| MdYUCCA8a-promoter-Luc | ggggtaccAGAAAACAGTACGGAAGCAGTTTTT | ggtggactcctcttaaagcttGCTTATGATCAAAGAAGTAGTTGCTCA | Clone |
| MdYUCCA10a-promoter-Luc | ggggtaccTGTGCACGTTGTTCTTGTCTC | ggtggactcctcttaaagcttTTCACTCGTGTGTATACTGTTTTG | Clone |
| MdYUCCA8a- promoter -pCAMBIA1381-GUS | gcgccgaattcccggggatccAGAAAACAGTACGGAAGCAGTTTTT | ggtggactcctcttaaagcttGCTTATGATCAAAGAAGTAGTTGCTCA | Clone |
| MdYUCCA10a- promoter -pCAMBIA1381-GUS | ggtggactcctcttaaagcttGTGCACGTTGTTCTTGTCTC | ggtggactcctcttaaagcttTTCACTCGTGTGTATACTGTTTTG | Clone |
| MdYUCCA8-CDS-1301 | acgggggactcttgaccatggATGGAGAACTTGTTTCGATTAGCTG | tagaaatttaccctcagatctGAACTGGGAAATCATCTTCTATGGC | Clone |
